# The Diagnostic Dilemma of Urothelial Tissue Fragments in Urinary Tract Cytology Specimens

**DOI:** 10.3390/diagnostics12040931

**Published:** 2022-04-08

**Authors:** Derek B. Allison, M. Lisa Zhang, Poonam Vohra, Christopher J. VandenBussche

**Affiliations:** 1 Department of Pathology and Laboratory Medicine, Lexington, KY 40536, USA; derek.allison@uky.edu; 2 Department of Urology, University of Kentucky College of Medicine, Lexington, KY 40536, USA; 3 Department of Pathology, Massachusetts General Hospital, Boston, MA 02114, USA; mlzhang@mgh.harvard.edu; 4 Departments of Pathology and Laboratory Medicine, University of California San Francisco, San Francisco, CA 94143, USA; poonam.vohra@ucsf.edu; 5 Departments of Pathology and Oncology, The Johns Hopkins University School of Medicine, Baltimore, MD 21205, USA

**Keywords:** urine, tissue fragments, bladder cancer, low grade urothelial carcinoma, urothelial

## Abstract

Since the release of The Paris System for Reporting Urinary Cytology (TPS), the assessment of urine cytology specimens has primarily focused on the detection of high-grade urothelial carcinoma (HGUC) and carcinoma in situ (CIS). Fortunately, the malignant cells in these lesions tend to be loosely cohesive, resulting in the natural exfoliation of individual malignant cells into the urine. However, HGUC/CIS lesions occasionally exfoliate larger fragments which can be difficult to assess due to cellular overlap and fragment three-dimensionality. Furthermore, reactive benign urothelial fragments and fragments from low-grade urothelial neoplasms (LGUN) may also be seen in urine specimens and contain atypical cytomorphologic features. As a result, the significance of urothelial tissue fragments (UTFs) is often unclear. Herein, we discuss the literature on UTFs before and after the implementation of TPS, as well as strategies to help overcome this diagnostic challenge.

## 1. Introduction

Since the release of The Paris System for Reporting Urinary Cytology (TPS), the assessment of urine cytology specimens has primarily focused on the detection of high-grade urothelial carcinoma (HGUC) and carcinoma in situ (CIS) [1,2,3]. Fortunately, the malignant cells in these lesions tend to be loosely cohesive, resulting in the natural exfoliation of individual malignant cells into the urine. However, HGUC/CIS lesions occasionally exfoliate larger fragments which can be difficult to assess due to cellular overlap and fragment three-dimensionality. Furthermore, reactive benign urothelial fragments and fragments from low-grade urothelial neoplasms (LGUN) may also be seen in urine specimens and contain atypical cytomorphologic features [4]. As a result, the significance of urothelial tissue fragments (UTFs) is often unclear. Herein, we discuss the literature on UTFs before and after the implementation of TPS as well as strategies to help overcome this diagnostic challenge.

## 2. Urothelial Tissue Fragments Prior to the Implementation of TPS

The presence of urothelial tissue fragments (UTFs) in urinary tract cytology (UTC) has been noted since Dr. Papanicolaou first described the examination of urine sediment specimens to detect urothelial tract neoplasms in the 1940s [5,6]. In these reports, Dr. Papanicolaou recommended that a definitive diagnosis of malignancy should be made based on the presence of “clusters” of cells with malignant features, though these features were not described in detail. Despite numerous reports over the last several decades, the literature is filled with conflicting results regarding the diagnostic meaning of UTFs in UTC [7,8,9,10,11,12,13]. This lack of clarity is partly due to a lack of a standardized definition for what constitutes a UTF and is further confounded by changes in the diagnostic criteria and terminology for urothelial carcinomas. Finally, prior to The Paris System for Reporting Urinary Cytology, many of these reports merely correlated the presence of UTF with any type of urothelial neoplasia on follow-up. Given that the current goal of UTC is to identify HGUC, many of these reports are simply not translatable to the current era of practice.

According to Dr. Frost in 1972, UTFs should refer to a collection of cells that arise from a common precursor and grow together in vivo [14]. As a result, UTFs should be comprised of cohesive cells that may be arranged in a flat sheet or in a three-dimensional configuration [15]. In contrast, “cell clusters”, “cell aggregates”, or “cell groups” are more loosely arranged, with spaces between cells and may be the result of concentration artifacts or centrifugation procedures [16]. In prior reports, this distinction is not always made, and one can imagine that the reproducibility of distinguishing a UTF from a cluster due to a preparation artifact is likely not high. Interestingly, Goldstein et al. found that flat sheets and collared, three-dimensional groups were more commonly seen in voided urine specimens with negative biopsies than in cases showing invasive carcinoma on follow-up [17]. In contrast, irregular, three-dimensional cell groups were more commonly seen in cases showing invasive carcinoma but were overall non-specific. In addition, they reported that cell groups of any type were found in similar proportions in cases with negative biopsies versus cases with low-grade papillary carcinoma (according to 1973 WHO criteria). As a result, the authors concluded that the presence of any type of cell group in voided urine specimens has little diagnostic utility. However, a similar study was subsequently conducted by Nasuti et al. and showed that the presence of UTFs was highly significant and correlated well with neoplastic change [16]. These authors suggested that the main difference in their study was due to the cytopreparation technique, suggesting that centrifugation creates artifactual UTFs not observable in specimens processed using the Millipore filter technique. Although the significance of UTFs seemed to be high in the study by Nasuti, they were still present in a subset of cases with benign findings on biopsy. Both of these studies, however, really lack detailed assessments of the associated cytomorphologic features beyond the architecture of the UTFs.

There are several reasons why UTFs may be in a UTC specimen. UTFs are commonly encountered in instrumented specimens due to the shearing forces of the procedure that causes disruption of the urothelial lining. This phenomenon also occurs in pelvic washing specimens, which often yield large sheets and tissue fragments of benign mesothelial cells. In addition, any trauma, such as infection or the presence of renal stones, can produce shedding of the urothelial lining to yield UTFs. This finding is true even in voided urines for which UTFs have also been correlated with abdominal palpation, prostate or rectal manipulation, and jogging prior to collection [18]. In 1978, Dr. Koss reported a classification scheme that corresponded to the three-tiered grading system (low-grade, intermediate-grade, high-grade) that had recently been established in the 1973 World Health Organization (WHO) classification system [19,20]. Dr. Koss noted that bland clusters/fragments with or without nuclear elongation and nuclear palisading are commonly observed in inflammatory conditions, urothelial papillomas, and low-grade papillary carcinomas. As a result, UTFs were interpreted to be a non-specific finding. More specifically, according to Dr. Koss, it was the cytomorphologic features that predicted intermediate-grade and high-grade papillary carcinomas, which included nuclear hyperchromasia (the most important feature), abnormal chromatin, and irregular nuclear contours.

In 1984, Murphy et al. suggested a separate classification system and offered guidance in making a diagnosis of a LGUN (including papilloma and low-grade papillary carcinoma according to the 1973 WHO criteria), noting that these cases are often more cellular and contain papillary and loose clusters of cells that lack significant atypical features [8]. However, the authors conceded that these features often overlap with reactive changes, and as a result, there will be many false-positive diagnoses. Like Dr. Koss’s study, this report shows the overlapping cytomorphologic features between a LGUN and non-neoplastic changes. They further showed that diagnostic accuracy is mainly limited to higher-grade carcinomas (intermediate-grade and high-grade according to the 1973 WHO criteria) and relies on the presence or absence of certain cytomorphologic features and not on the presence of UTFs. As a result, Murphy et al. proposed a two-tiered classification scheme of low-grade and high-grade carcinomas for UTC.

In 1998, the WHO and the International Society of Urology Pathology updated the classification of urinary tract cancers [21]. In this new system, papillary tumors with cytomorphologic features that overlap with normal urothelium were labeled as benign urothelial papillomas or papillary urothelial neoplasm of low malignant potential (PUNLMP)—the difference being mainly architectural features. Importantly, a subset of cases previously classified as low-grade papillary carcinoma based on the 1973 WHO classification system would meet the criteria for PUNLMP according to these guidelines and under the current 2016 WHO classification system; the remaining cases would be categorized as either low-grade or high-grade, based mainly on nuclear atypia. Low-grade papillary urothelial carcinoma shows abnormal polarity, as well as subtle but definitive cytologic atypia, including small variations in nuclear size, shape, and chromatin texture. One can imagine how difficult it would be to detect subtle atypia and polarity abnormalities in UTC given the lack of association with a large, intact papillary structure that would be apparent in a biopsy or resection specimen. In contrast, HGUCs contain completely disorganized cells that show a spectrum of pleomorphism and significant nuclear hyperchromasia, clumped chromatin, prominent nucleoli, and abundant mitoses. Fortunately, these cytomorphologic features are something that can be assessed in UTC.

Since this two-tiered updated system, the literature has shown that the performance of UTC in detecting HGUC is high, whereas its sensitivity and specificity in detecting a LGUN (which includes papilloma, PUNLMP, and low-grade papillary urothelial carcinoma) is poor [22,23,24,25]. As previously mentioned, much of the reason for this poor performance is the lack of or the limited atypia present in LGUN. Therefore, some authors have argued that the diagnosis should be reserved only for cases with an unequivocal three-dimensional tissue fragment with a central fibrovascular core, coupled with bland cytologic features [26,27]. Since the presence of true papillary UTFs is extremely rare in UTC, this diagnosis is not made with any routine frequency. In one report, other features that have been associated with LGUN include high cellularity, single cells, UTF, cytoplasmic tails with eccentric nuclei, mild hyperchromasia, mild pleomorphism, and nuclear contour irregularity; however, many of these changes were more pronounced in urothelial washing specimens than in voided urines and were only identified upon retrospective review [4]. In a more recent study just prior to TPS, Onur et al. found that benign-appearing UTFs in voided urines were associated with a low rate of urothelial neoplasia [9]. More specifically, these bland UTFs were associated with urinary tract stones in 16.4% of the cases, LGUN in 3.6% of the cases, and HGUC in 0.7% of the cases. Furthermore, when compared to cases that were classified as benign based on UTC, the rates of LGUN and HGUC were similar at 2.3% and 0.7%, suggesting that the presence or absence of benign-appearing UTFs adds no diagnostic value. On a follow-up study, these authors also investigated voided urine specimens containing UTFs with atypia. In this report, 28.8% of the cases were associated with urinary tract stones, 1.2% were diagnosed with LGUN on follow-up, and 8.8% were diagnosed with HGUC on follow-up [10]. Although the rate of HGUC was higher in specimens with atypical UTFs than in specimens with benign-appearing UTFs, most cases were associated with no neoplastic disease. Although there is no description of what type of cytomorphologic features made the UTFs “atypical”, these studies together suggest that the mere presence of UTFs is not predictive of urothelial neoplasia. As a result, placing cases with benign-appearing or atypical UTFS in a worrisome category could trigger unnecessary concern, potentially leading to repeat cystoscopies that would not be clinically helpful or cost-effective. Consequently, urologists generally treat an “atypical” diagnosis the same as a benign diagnosis [11,28]. This practice comes from the American Urologic Association (AUA) guidelines, which addresses urinary cytology in several clinical scenarios, though nowhere is there a recommendation to change the diagnostic screening intervals for cystoscopy, UTC, or clinical management in response to an atypical diagnosis [29,30,31]. Since UTFs lacking high-grade features appear to be a poor predictor of neoplasia, and an atypical diagnosis does not change the current clinical management, most of these cases should be referred to as a negative diagnostic examinations.

## 3. Tissue Fragments Arising from Low-Grade Urothelial Neoplasms (LGUN)

Due to significant cytomorphologic overlap between low-grade urothelial neoplasms (LGUN) and non-neoplastic urothelium, TPS shifted the goal of urine cytology evaluation to focus on the identification of HGUC/CIS while de-emphasizing the detection of LGUN [32,33,34]. As such, the single required cytologic criterion for the diagnosis of LGUN put forth in TPS (applicable to both voided and instrumented urines) is stringent: three-dimensional (3D) cellular papillary clusters with fibrovascular cores containing central capillaries. Though many other features of LGUN have previously been reported, they are unreliable and can only be considered a forme fruste of definitive LGUN; thus, TPS recommends classifying any cases lacking papillary fragments with fibrovascular cores or other atypical features suggestive of HGUC under the Negative for HGUC (NHGUC) category [18].

Since the publication of TPS, many studies have reported the rates of identifying true papillary fragments, and some have compared the diagnostic rates of LGUN before vs. after the implementation of TPS. In a large cohort of biopsy-confirmed LGUN cases, Zhang et al. found that 88% of urine specimens (including voided and instrumented urines) had tissue fragments and 42% had few to many tissue fragments; however, only one case (0.7%) demonstrated true papillary fragments [4]. This study also found that instrumented urines/urinary washing (UW) specimens contained significantly more tissue fragments compared to both voided urines with histology-confirmed LGUN and benign UW specimens. In a follow-up study, UW specimens (but not voided urines) with AUTF were significantly associated with LGUN on follow-up biopsy compared to those without AUTF (22% vs. 9%) [35].

Cakir et al. evaluated 32 UW cases with histology-confirmed LGUC and found that 3D cellular papillary clusters without fibrovascular cores were significantly increased in LGUC vs. reactive urothelium (75% vs. 21%, *p* < 0.001); however, this feature was not found to be significant in a subsequent logistic regression analysis [36]. In contrast, papillary fragments with fibrovascular cores were only found in three cases (9%) of LGUC but not in any of the reactive cases. Bansal et al. evaluated 48 voided urine samples with histology-confirmed LGUC and found papillary fragments (defined as 3D papillary clusters of urothelial cells with significant nuclear overlapping, but not specified fibrovascular cores) in only two cases [37]. These three studies, taken together, suggest that LGUN is rarely associated with true papillary structures with fibrovascular cores on UTC. As a result, if this feature is required for a reliable diagnosis, UTC lacks sensitivity to detect LGUN. For tissue fragments without fibrovascular cores, TPS recommends interpreting the structures as benign UTF (classified as NHGUC) or atypical UTF (classified as Atypical Urothelial Cells or higher) depending on the absence or presence of cytologic atypia possessed by the cells making up the fragment.

Studies investigating the diagnostic rates of LGUN based on TPS criteria include those that report specificities of 100%, those with misclassified LGUN that are revealed to be HGUC on histologic follow-up, and those in which no cases met the criterion for categorization as LGUN. In the first group, Zare et al. [38] reported that 2/52 (4%) LGUN cases were correctly classified post-TPS (vs. 6% pre-TPS); Meilleroux et al. found that significantly fewer cases of LGUN were diagnosed post- vs. pre-TPS (0.9% vs. 1.8%), with sensitivities pre- and post-TPS of 31% and 10%, respectively, and specificities pre- and post-TPS of 96% and 100%, respectively [39]. Recently, Rohra et al. reported similar rates of LGUN pre- vs. post-TPS (0.2% vs. 0.17%), but also placed 4% of their cases into a separate category of “Negative for high-grade, cannot rule out low-grade urothelial neoplasm”, of which on follow-up, 67% were LGUN, 13% HGUC, and 20% negative [40]. The criteria for this category included 3D cellular clusters without fibrovascular cores.

However, three other studies reported cases classified as LGUN based on TPS criteria that showed HGUC on follow-up histology. In Ma et al., 6/143 (4.2%) cases were classified as LGUN, and follow-up histology showed LGUN in 4 cases and HGUC in 2 cases, resulting in a sensitivity of 12% and a specificity of 98% [41]. Roy et al. classified 13/97 (13%) cases as LGUN, and of those, 3 were HGUC on follow-up biopsy [42]. Lastly, Moulavasilis et al. classified 13 cases as LGUN, but 2 were HGUC on follow-up histology [43].

A handful of post-TPS studies focused on upper UTC also discussed the detection of LGUN in that specimen type [44]. Upper urinary tract specimens are procured by instrumentation and can result in the exfoliation of tissue fragments, which, similar to instrumented specimens of the lower urinary tract, makes the detection of LGUN more challenging [45,46]. McIntire et al. reported that the application of TPS improved the detection of LGUN cases, with the pre-TPS and post-TPS LGUN categories correctly identifying 15% vs. 30% of cases, respectively [47]. Simon et al. also found that the sensitivity of upper urinary tract cytology improved from 8% to 21% post-TPS for the detection of LGUN [48]. In contrast, Xing et al. reported that while 50% of LGUN were identified pre-TPS, none of the LGUN cases in their cohort were identified using TPS criteria, as they all lacked true papillary fragments with fibrovascular cores [49].

In summary, the implementation of the strict TPS criterion for LGUN results in the classification of only rare cases, leading to very low sensitivities but high specificities for the detection of LGUN. The majority of LGUN cases ending up in the NHGUC category is expected, as it is not crucial to distinguish LGUN from benign/reactive urothelium because, according to the AUA, UTC is not recommended in the initial evaluation of patients with microhematuria nor is it recommended to replace surveillance cystoscopy for patients with a history of bladder cancer. As a result, most of these patients will have had cystoscopy prior to or alongside UTC, which is very good at detecting LGUN, unlike urothelial CIS [29,30,31]. However, the rare instances in which HGUC was misdiagnosed as LGUN on UTC should be carefully re-evaluated to determine if the misclassification was due to (1) lack of sampling of a HGUC component or (2) the presence of more concerning atypia within individual cells despite the presence of papillary fragments with fibrovascular cores.

## 4. Cytomorphologic Assessment of Urothelial Tissue Fragments in Urinary Tract Specimens

**Figure 1:**
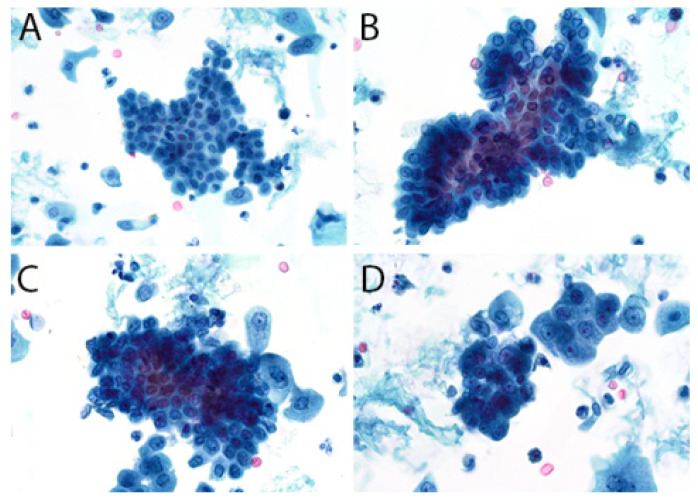
(**A**), The assessment of cytologic atypia is more easily performed when cellular overlap is minimal. In this sheet, it is easy to appreciate the uniformity of the nuclei. (**B**), The nuclei at the edges clearly have bland chromatin and lack hyperchromasia. In the center of the fragment, nuclear overlap can create a sense of nuclear hyperchromasia and elevated N/C ratio; in addition, chromatin pattern and nuclear contours are obscured. (**C**), Many of the cells in this fragment appear to have elevated N/C ratios and contain nuclear contour irregularities. Their benign nature may be suggested by the overlying umbrella cells, seen at the right-hand side of the fragment. However, umbrella cells may also be seen on the surface of LGUN, CIS, and even HGUC; thus, this feature is not reassuring on its own. (**D**), Two small fragments containing predominantly umbrella cells. They can be identified by their abundant and granular cytoplasm; however, the cells in the fragment on the left are overlapping and appear to have elevated N/C ratios. In these instances, the distinct chromatin pattern of umbrella cells can be useful: a single nucleolus together with a condensed rim of chromatin around the nuclear border.

**Figure 2:**
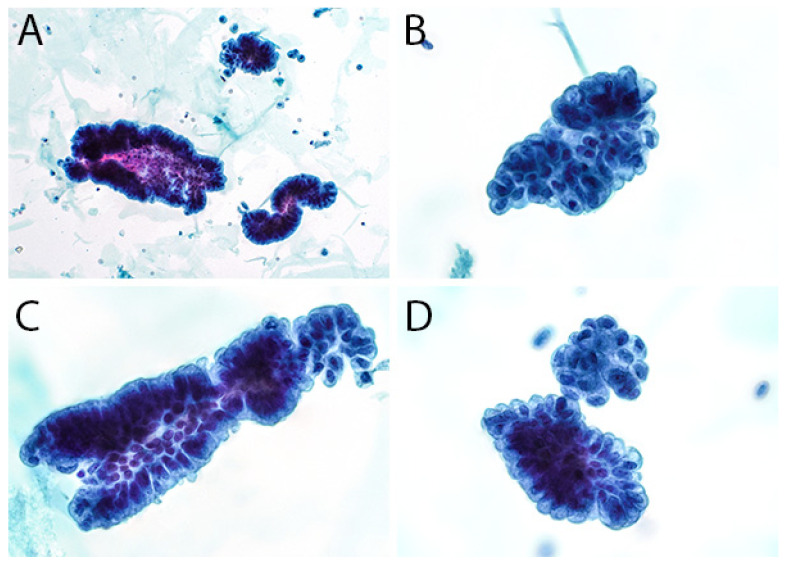
(**A**–**D**), Bland-appearing urothelial tissue fragments. While these fragments have a papillary appearance, they lack fibrovascular cores and thus may represent fragments of benign urothelium. The nuclei are oval-shaped and similar in size; their chromatin pattern is uniform, with nuclei containing 1–2 small chromocenters. Some mild irregularities in the nuclear contour can be appreciated. The cells at the edges of the fragment do not have elevated N/C ratios, with their cytoplasm forming a “collarette”.

**Figure 3:**
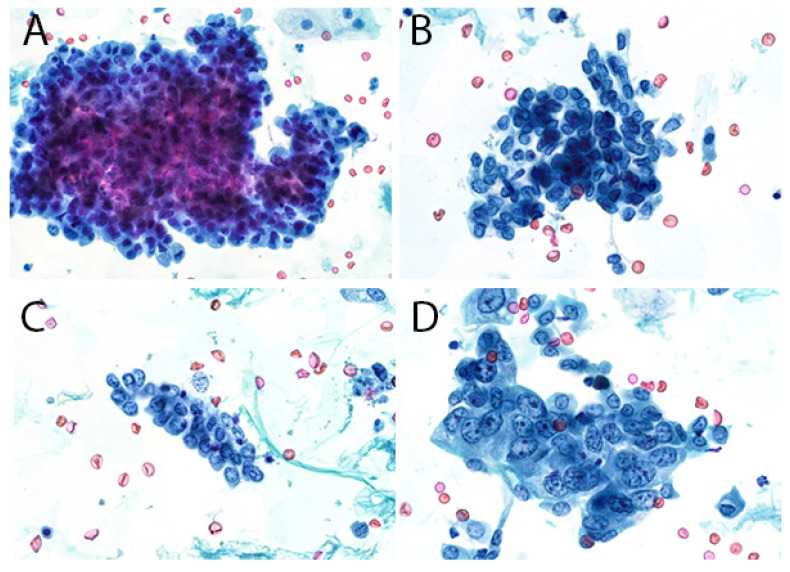
Fragments from a specimen containing high-grade urothelial carcinoma. (**A**), At low magnification, the nuclei appear dark and disorganized within the fragment. Assessment at higher magnification is required. (**B**), The nuclei in this fragment have high N/C ratios and nuclear contour irregularities. However, the nuclei are around the same size, and the chromatin pattern is bland. It contains sufficient atypia to consider the Atypical Urothelial Cells category. (**C**), The cells in this fragment have high N/C ratios and contain irregularly distributed chromatin clumps. There is a small but noticeable variation in nuclear size (2:1). The chromatin is paradoxically hypochromatic. (**D**), This fragment contains cells with overtly malignant features. The nuclear contours are markedly irregular, and there is significant variation in nuclear size (4:1). The chromatin forms randomly distributed clumps.

**Figure 4:**
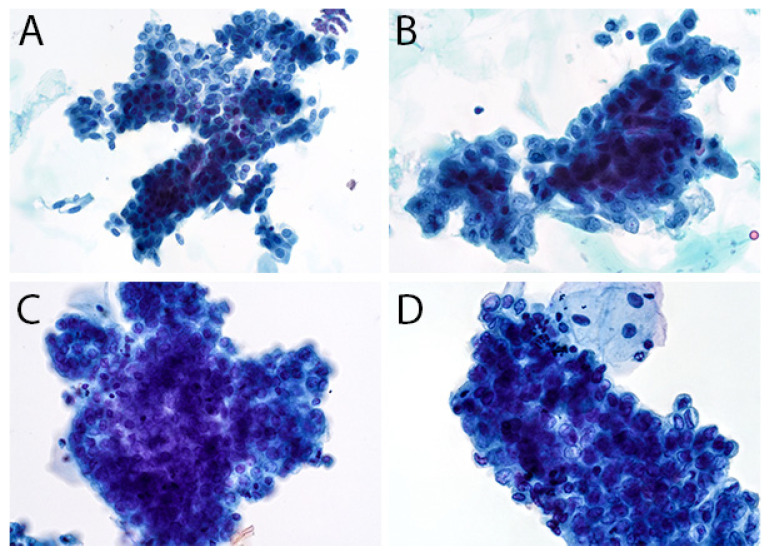
(**A**,**B**), Two tissue fragments from a specimen diagnosed as AUC with benign follow-up. (**A**), The nuclei appear enlarged, some with elevated N/C ratios, but are similar in size and have regular nuclear contours. Several columnar cells are attached to the edges; cells with such morphology can be seen in patients with cystitis glandularis but also low-grade urothelial neoplasms. (**B**), The nuclei in a second fragment have some nuclear size variation and nuclear membrane irregularities. However, the chromatin is bland, with nuclei containing 1–2 small chromocenters; furthermore, many of the cells at the edges lack elevated N/C ratios. (**C**,**D**), Two tissue fragments from a specimen diagnosed as SHGUC with malignant follow-up. (**C**), There is significant nuclear overlap in this fragment, which makes a morphologic assessment challenging. The nuclei appear mostly the same size. (**D**), By comparison, this second fragment has more atypical features, with more significant anisonucleosis and nuclear contour irregularities. When assessed at the edges of the fragments, the N/C ratios appear elevated. The chromatin lacks the coarse, clumpy pattern and instead appears paradoxically hypochromatic.

The second edition of TPS expanded the discussion on UTFs and included additional photographs illustrating the differences between atypical UTFs and benign UTFs. Differentiating between benign urothelium and LGUN fragments is not a critical issue in TPS, as the second edition places both entities under the NHGUC category (Figure 1 and Figure 2) [18]. Prior to this, the first edition allowed LGUN to be diagnosed in its own diagnostic category, so long as the cytologic specimen contained true papillary urothelial fragments lacking high-grade atypia, as well as the identification of a papillary lesion on cystoscopy. While a fibrovascular core is important for suggesting the presence of LGUN, atypical cytologic features are used to assess the risk for HGUC and establish a diagnosis of *Atypical Urothelial Cells* (AUC), *Suspicious for HGUC* (SHGUC), or *HGUC* (Figure 3 and Figure 4) [50,51,52,53]. In general, the same cytomorphologic features used to classify individual urothelial cells can be used to classify urothelial cells in tissue fragments. However, challenges arise in assessing four important cytomorphologic features in tissue fragments. Due to cellular overlap and fragment three-dimensionality, the nuclei may appear more hyperchromatic than nuclei in singly dispersed cells; the nuclear-to-cytoplasmic ratios may appear falsely elevated due to obscured cytoplasmic boundaries; and the chromatin pattern and nuclear membranes may be obscured. Additionally, cells within fragments are physically attached, resulting in forces to be transferred between cells. These forces can cause irregularities in the cell and nuclear shape; thus, some increase in nuclear contour irregularity should be expected. These issues not only confound the evaluation by the human eye, but also impact digital image analysis. Furthermore, digital image analysis of cytologic specimens is currently performed on one Z-stack, whereas tissue fragments are often three-dimensional and contain cells at multiple levels of focus.

The assessment of UTFs should therefore be performed with a careful consideration of the above limitations. The central areas of fragments and/or areas of significant cellular overlap should be assessed with a greatly increased threshold for identifying atypia. The edges of tissue fragments can allow for a better assessment of nuclear hyperchromasia and chromatin pattern. Cytoplasm may be seen at the edge of fragments and help identify cells with low N/C ratios. However, if the nucleus is at the fragment edge, it may be difficult to assess a cell’s true N/C ratio [54,55]. Finally, mild nuclear contour irregularities should be expected in benign tissue fragments. While not a primary feature in TPS, prominent anisonucleosis can help differentiate between atypical and benign tissue fragments, as benign urothelium and many LGUN lesions tend to have monomorphic nuclei (Table 1). This feature may be helpful in instances where the nuclei can be clearly identified within a tissue fragment. In some instances, LGUN lesions may contain cells with more pleomorphic nuclei, resulting in their classification into the AUC or SHGUC categories [56].

There have been no rigorous comparative studies of the impact of the preparation type (e.g., Cytospin, ThinPrep, SurePath) on the presence or morphology of urothelial tissue fragments; numerous studies have demonstrated that SurePath preparations in various specimen types tend to retain a greater number of medium-to-large sized tissue fragments compared to ThinPrep preparations [57,58,59]. It is likely that urinary cytology specimens also demonstrate this phenomenon.

## 5. Urothelial Tissue Fragments in Cell Block Preparations

**Figure 5:**
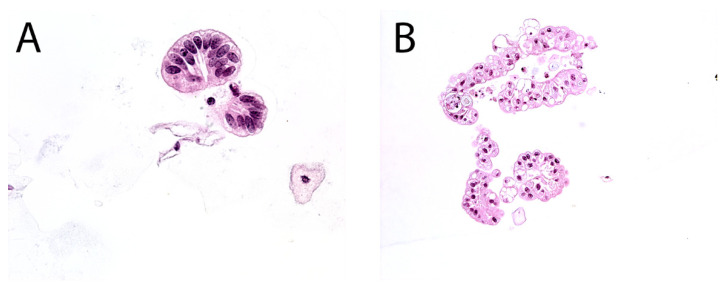
Benign urothelial tissue fragments in cell block preparations. (**A**,**B**) Examples of benign-appearing urothelial tissue fragments demonstrating bland, uniform nuclei with fine chromatin and moderately abundant cytoplasm from patients who lacked urothelial neoplasia on follow-up.

**Figure 6:**
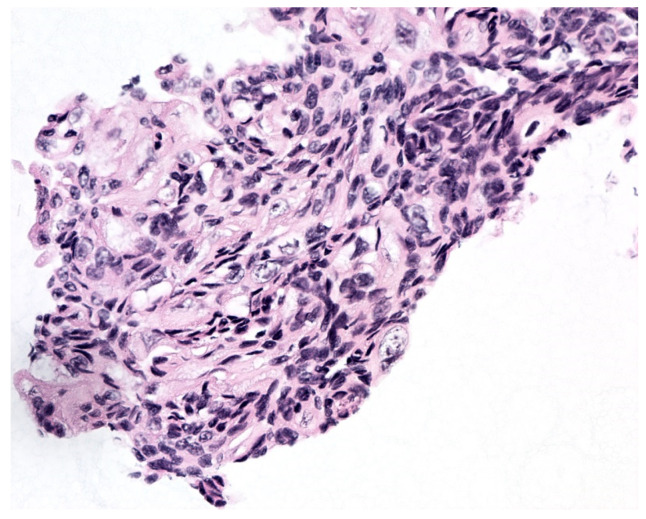
Stone atypia in a cell block preparation. The fragment demonstrates urothelial cells with irregular nuclear contours and hyperchromasia due to the overlapping of nuclei in a patient with a history of recurrent bladder stones. However, no papillary formations can be seen, and cells in the upper left corner show bland chromatin distribution and lack of hyperchromasia. Given the history of stones and focal cytologic atypia, the findings are negative for high-grade urothelial carcinoma.

The preparation of CBs from selective UTC specimens may help better characterize UTFs, as cell block preparations eliminate issues with three-dimensionality and overlap associated with conventional cytologic preparations (Figure 5 and Figure 6) [60]. CBs can be prepared using a variety of techniques; regardless of the technique used, disaggregated cells and tissue fragments are ultimately concentrated into a pellet that can be processed like small tissue biopsies. Techniques vary due to the use of different fixatives (formalin, alcohol) and congealing agents (HistoGel, agar, plasma and thrombin, collodion bag) used to make a cell pellet. In most instances, once a pellet is formed, the sample is placed in a tissue cassette and processed as a histology specimen. Generally, CBs have been underutilized in urine cytology due to scant diagnostic material and increased turnaround time and cost [61,62]. As per the College of American Pathologists (CAP) Cytopathology Committee survey regarding laboratory processing and reporting of urine cytology specimens, 23.3% of laboratories reported the use of CB preparation, out of which most laboratories (84.4%) reported the selective use of CB, and only 15.7% utilized cell blocks on all urine cytology cases [63]. Therefore, there is a paucity of published data on the performance of CB in urine cytology. A few recently published studies reported that a CB preparation can contribute to a final diagnosis in a range of UTC cases (including benign, atypical, HGUC, and metastatic disease), as CBs are a valuable resource for ancillary testing [49,61,62,64,65,66,67].

**Figure 7:**
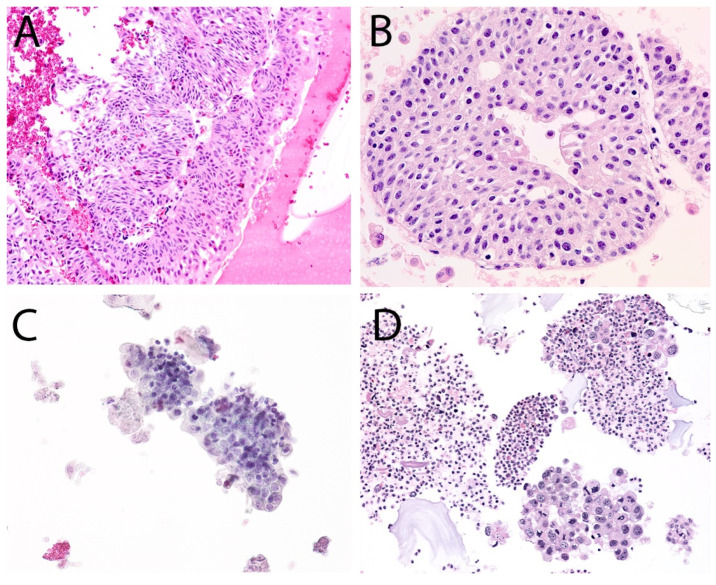
Examples of low-grade urothelial carcinoma (**A**,**B**) and high-grade urothelial carcinoma (**C**,**D**) in cell block preparations. Cell block preparations may allow for a better assessment of atypical cytologic features in tissue fragments that are otherwise obscured in cytologic preparations. Here, the high-grade urothelial carcinoma nuclei (**C**,**D**) are enlarged and more pleomorphic, whereas the low-grade urothelial carcinoma nuclei (**A**,**B**) are monotonous, and the cells have lower nuclear-to-cytoplasmic ratios.

Although the identification of LGUN is not encouraged by TPS, CBs have been shown to improve the sensitivity of urine cytology for the diagnosis LGUN because they increase the likelihood of visualizing a papillary architecture with fibrovascular cores [49,61,62,64,66,68]. In addition, washings have more likelihood to show small tissue fragments in CBs, making the diagnosis of LGUN easier as compared to voided urine specimens (Figure 7) [4]. Dantey et al. reported that the judicious use of CBs decreased the atypical rate [62]. The utility of CB in cases of HGUC has been shown to be mainly limited to providing material for IHC and to support a urothelial origin [61]. In some cases, CBs can highlight the papillary architecture of HGUC.

**Figure 8:**
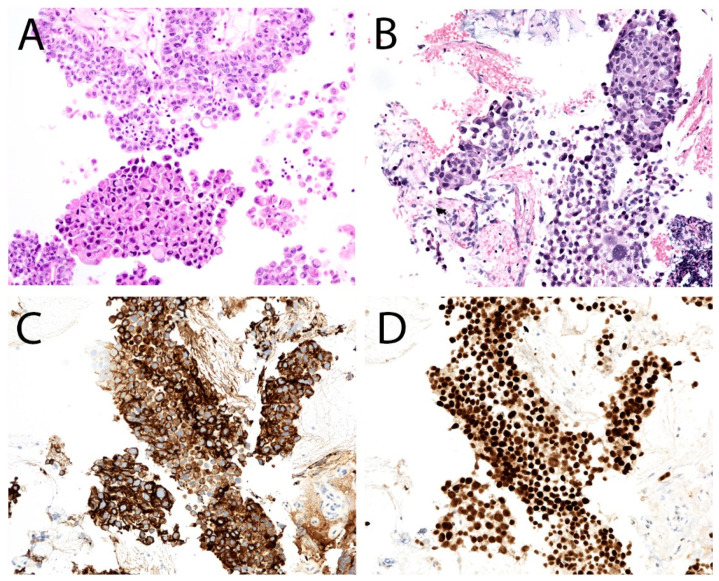
Two examples of high-grade urothelial carcinoma in cell block preparations (**A**,**B**). Example A demonstrate the papillary architecture of a high-grade urothelial carcinoma. For the second example, diffuse staining with CK20 (**C**) and p53 (**D**) supported the diagnosis, highlighting the utility of cell block preparations when used together with immunohistochemistry.

The utility of CBs is recognized not only in capturing tissue fragments for diagnosis but also in ancillary testing, including IHC for confirming the diagnosis of HGUC or distinguishing nonurothelial lesions such as metastatic tumors (Figure 8). This is particularly important in laboratories that do not have IHC protocols for non-CB cytology specimens [64]. One study reported performing a triple stain (CK20, p53, and CD44) in atypical cytology samples to distinguish HGUC from its mimics, including degenerative/reactive atypia. In this study, a positive triple stain (CK20+, p53+, and CD44+) was found to have a sensitivity of 95% and a specificity of 78.3% for detecting HGUC [69].

Recent studies have indicated that CB preparations might be helpful in upper tract specimens where biopsy may not be possible. CBs from ureteral washings often contain intact fragments and large clusters of urothelial cells, sometimes even larger groups of cells than actual ureteral biopsy specimens. Therefore, the use of CBs in ureteral washing specimens can aid in the correct diagnosis of these challenging specimens [70]. In addition, CB preparations of ureteral microbiopsies have been reported to offer greater sensitivity in the detection of urothelial carcinoma by minimizing the loss of cellular material compared with conventional histologic processing, as the tissue fragments from upper urinary tract biopsies may not survive conventional processing by surgical pathology [71].

CB processing is not standardized, and different processing techniques (HistoGel/agar, centrifugation sediment, collodion bag or Cellient) are utilized for CB preparation in UTC across laboratories [72,73]. Reasons or triggers to make a CB also vary among institutions. CBs prepared from samples with visible urine sediment/good pellets after centrifugation provide better tissue architecture as compared to those with low cellularity and/or without visible sediment [61,62,65,68]. Other guiding factors for CB preparation include specimens collected by cystoscopy, suspected lesions of non-urothelial origin, or cases with scant atypical cells/clusters [62]. Brisuda et al. reported that adequate cellularity in urine CBs was also found in cases with positive urine cytology, female sex, and positive leukocyturia [65].

## 6. Conclusions

While both editions of TPS provide a framework for assessing and reporting urothelial tissue fragments, few studies have examined UTF morphology since the arrival of the first edition. Generally, TPS recommends the assessment of individual cells within fragments using the same criteria for single urothelial cells. However, this guidance has not been rigorously assessed in the literature. Additional studies of UTF morphology are needed to better guide their assessment in the context of TPS. In addition, additional prospective studies of cell blocks and ancillary studies are encouraged to determine whether these may provide useful adjuncts to conventional cytomorphology for classifying specimens containing atypical UTFs [67,74,75].

## Figures and Tables

**Table 1:** Differential diagnoses associated with urothelial tissue fragments based on the morphology seen, and helpful features that may help distinguish between diagnoses.

Morphology	Differential Diagnosis	Helpful Distinguishing Features
Bland-appearing urothelial tissue fragments (BUTFs) lacking fibrovascular cores Note: Regardless of whether the BUTFs in question represent LGUN or benign urothelium, they would be classified under the NHGUC category according to TPS.	Benign urothelium	Umbrella cells may be attached to intermediate cells in benign fragments
	LGUN	Singly dispersed cells with cytoplasmic tails (cercariform cells) may be present in washing specimens Cellular population of monomorphic cells, in washing specimens
Bland-appearing urothelial tissue fragments with fibrovascular cores Note: While UTFs with fibrovascular cores lacking atypical features usually represent LGUN, some HGUCs can be seen as a monotonous population of cells and/or have paradoxically hypochromatic nuclei. If HGUC cannot be excluded, an indeterminate TPS category can be used.	LGUN	Monomorphic population of cells Singly dispersed cells with cytoplasmic tails (cercariform cells) may be present in washing specimens N/C ratios are usually 0.5 or less Bland chromatin with few chromocenters
	HGUC	Marked anisonucleosis and pleomorphism High N/C ratios Irregular nuclear contours Coarse/clump irregular chromatin pattern Single atypical cells usually dispersed in the background in voided urine specimens (e.g., malignant cells rarely only seen in fragments) Cercariform forms may be seen but only in small proportions Occasionally paradoxical hypochromasia with irregular chromatin pattern
Atypical-appearing urothelial tissue fragments (AUTFs) lacking fibrovascular cores Note: Some LGUN lesions may produce cells with increased pleomorphism than is typically seen. Thus, some AUTFs classified into indeterminate categories may end up representing LGUN.	Stone atypia	Atypical fragments should not be seen in great numbers Small, dark nuclei with irregular contours Chromatin may be difficult to assess due to hyperchromasia but should not demonstrate a coarse/clumpy irregular pattern. N/C ratios may be elevated due to small cell size
	LGUN	Bland chromatin with few chromocenters, lacking hyperchromasia Oval-shaped nuclei with regular contours Cellular population of monomorphic cells, in washing specimens
	HGUC	Larger, pleomorphic cells Coarse/clumpy irregular chromatin High N/C ratios in larger cells Single atypical cells usually dispersed in the background (e.g., malignant cells rarely only seen in fragments)
Atypical-appearing urothelial tissue fragments (AUTFs) with fibrovascular cores Note: Some LGUN lesions may produce cells with increased pleomorphism than is typically seen. Thus, some AUTFs classified into indeterminate categories may end up representing LGUN.	LGUN	Bland chromatin with few chromocenters Lack hyperchromasia Monomorphic population of cells
	HGUC	Coarse/clumpy irregular chromatin Increased pleomorphism and anisonucleosis Occasionally paradoxical hypochromasia with irregular chromatin pattern

## References

[1] Barkan G.A., Wojcik E.M., Nayar R., Savic-Prince S., Quek M.L., Kurtycz D.F., Rosenthal D.L. (2016). The Paris system for reporting urinary cytology: The quest to develop a standardized terminology. Acta Cytol..

[2] VandenBussche C.J. (2016). A review of the Paris system for reporting urinary cytology. Cytopathology.

[3] Wojcik E.M., Kurtycz D.F., Rosenthal D.L. (2022). We’ll always have Paris the Paris system for reporting urinary cytology 2022. J. Am. Soc. Cytopathol..

[4] Zhang M.L., Rosenthal D.L., VandenBussche C.J. (2016). The cytomorphological features of low-grade urothelial neoplasms vary by specimen type. Cancer Cytopathol..

[5] Papanicolaou G.N., Marshall V.F. (1945). Urine sediment smears as a diagnostic procedure in cancers of the urinary tract. Science.

[6] Papanicolaou G.N. (1947). Cytology of the urine sediment in neoplasms of the urinary tract. J. Urol..

[7] Owens C.L., Vandenbussche C.J., Burroughs F.H., Rosenthal D.L. (2013). A review of reporting systems and terminology for urine cytology. Cancer Cytopathol..

[8] Murphy W.M., Soloway M.S., Jukkola A.F., Crabtree W.N., Ford K.S. (1984). Urinary cytology and bladder cancer. The cellular features of transitional cell neoplasms. Cancer.

[9] Onur I., Rosenthal D.L., VandenBussche C.J. (2015). Benign-appearing urothelial tissue fragments in noninstrumented voided urine specimens are associated with low rates of urothelial neoplasia. Cancer Cytopathol..

[10] Onur I., Rosenthal D.L., VandenBussche C.J. (2015). Atypical urothelial tissue fragments in noninstrumented voided urine specimens are associated with low but significantly higher rates of urothelial neoplasia than benign-appearing urothelial tissue fragments. Cancer Cytopathol..

[11] Allison D.B., Kates M., VandenBussche C.J. (2022). Indeterminate atypia in urinary tract cytology: Does it really matter?. Diagn. Cytopathol..

[12] Rosenthal D.L., VandenBussche C.J., Burroughs F.H., Sathiyamoorthy S., Guan H., Owens C. (2013). The Johns Hopkins Hospital template for urologic cytology samples: Part I—Creating the template. Cancer Cytopathol..

[13] VandenBussche C.J., Sathiyamoorthy S., Owens C.L., Burroughs F.H., Rosenthal D.L., Guan H. (2013). The Johns Hopkins Hospital template for urologic cytology samples: Parts II and III—Improving the predictability of indeterminate results in urinary cytologic samples: An outcomes and cytomorphologic study. Cancer Cytopathol..

[14] Frost J.K. (1972). Concepts Basic To General Cytopathology.

[15] Deshpande V., McKee G.T. (2005). Analysis of atypical urine cytology in a tertiary care center. Cancer.

[16] Nasuti J.F., Fleisher S.R., Gupta P.K. (2001). Significance of tissue fragments in voided urine specimens. Acta Cytol..

[17] Goldstein M.L., Whitman T., Renshaw A.A. (1998). Significance of cell groups in voided urine. Acta Cytol..

[18] VandenBussche C.J., Chandra A., Heymann J.J., McCroskey Z., Owens C.L., Schubert P.T., Wang Y.H. (2022). Negative for high-grade urothelial carcinoma (NHGUC). The Paris System for Reporting Urinary Cytology.

[19] Koss L.G., Bartels P.H., Sychra J.J., Wied G.L. (1978). Diagnostic cytologic sample profiles in patients with bladder cancer using TICAS system. Acta Cytol..

[20] Bartels P.H., Koss L.G., Sychra J.J., Wied G.L. (1978). Indices of cell atypia in urinary tract cytology. Acta Cytol..

[21] Epstein J.I., Amin M.B., Reuter V.R., Mostofi F.K. (1998). The World Health Organization/International Society of Urological Pathology consensus classification of urothelial (transitional cell) neoplasms of the urinary bladder. Am. J. Surg. Pathol..

[22] Bastacky S., Ibrahim S., Wilczynski S.P., Murphy W.M. (1999). The accuracy of urinary cytology in daily practice. Cancer.

[23] Chu Y.C., Han J.Y., Han H.S., Kim J.M., Suh J.K. (2002). Cytologic evaluation of low grade transitional cell carcinoma and instrument artifact in bladder washings. Acta Cytol..

[24] Garbar C., Mascaux C., Wespes E. (2007). Is urinary tract cytology still useful for diagnosis of bladder carcinomas? A large series of 592 bladder washings using a five-category classification of different cytological diagnoses. Cytopathology.

[25] Cowan M.L., Rosenthal D.L., VandenBussche C.J. (2017). Improved risk stratification for patients with high-grade urothelial carcinoma following application of the Paris system for reporting urinary cytology. Cancer Cytopathol..

[26] Renshaw A.A., Nappi D., Weinberg D.S. (1996). Cytology of grade 1 papillary transitional cell carcinoma. A comparison of cytologic, architectural and morphometric criteria in cystoscopically obtained urine. Acta Cytol..

[27] VandenBussche C.J., Allison D.B., Gupta M., Ali S.Z., Rosenthal D.L. (2018). A 20-year and 46,000-specimen journey to Paris reveals the influence of reporting systems and passive peer feedback on pathologist practice patterns. Cancer Cytopathol..

[28] Gupta M., VandenBussche C., Bivalacqua T. (2018). Urinary cytology and the Paris system for reporting urinary cytology: Implications for urological management. Cytopathology.

[29] Chang S.S., Boorjian S.A., Chou R., Clark P.E., Daneshmand S., Konety B.R., Pruthi R., Quale D.Z., Ritch C.R., Seigne J.D. (2016). Diagnosis and treatment of non-muscle invasive bladder cancer: AUA/SUO guideline. J. Urol..

[30] Barocas D.A., Boorjian S.A., Alvarez R.D., Downs T.M., Gross C.P., Hamilton B.D., Kobashi K.C., Lipman R.R., Lotan Y., Ng C.K. (2020). Microhematuria: Aua/sufu guideline. J. Urol..

[31] Chang S.S., Bochner B.H., Chou R., Dreicer R., Kamat A.M., Lerner S.P., Lotan Y., Meeks J.J., Michalski J.M., Morgan T.M. (2017). Treatment of non-metastatic muscle-invasive bladder cancer: AUA/ASCO/ASTRO/SUO guideline. J. Urol..

[32] McCroskey Z., Pambuccian S.E., Kleitherms S., Antic T., Cohen M.B., Barkan G.A., Wojcik E.M. (2015). Accuracy and interobserver variability of the cytologic diagnosis of low-grade urothelial carcinoma in instrumented urinary tract cytology specimens. Am. J. Clin. Pathol..

[33] Rosenthal D.L., Wojcik E., Kurtycz D.F. (2015). The Paris System for Reporting Urinary Cytology.

[34] Kurtycz D.F.I., Barkan G.A., Pavelec D.M., Rosenthal D.L., Wojcik E.M., VandenBussche C.J., Mangiulli K., Olson M.T. (2018). Paris Interobserver Reproducibility Study (PIRST). J. Am. Soc. Cytopathol..

[35] Zhang M.L., Rosenthal D.L., VandenBussche C.J. (2017). Upper urinary tract washings outperform voided urine specimens to detect upper tract high-grade urothelial carcinoma. Diagn. Cytopathol..

[36] Cakir E., Kucuk U., Pala E.E., Sezer O., Ekin R.G., Cakmak O. (2017). Cytopathologic differential diagnosis of low-grade urothelial carcinoma and reactive urothelial proliferation in bladder washings: A logistic regression analysis. APMIS.

[37] Bansal S., Pathuthara S., Joseph S., Dighe S., Menon S., Desai S.B. (2021). Is diagnosis of low-grade urothelial carcinoma possible in urine cytology?. J. Cytol..

[38] Zare S., Mirsadraei L., Reisian N., Liao X., Roma A., Shabaik A., Hasteh F. (2018). A single institutional experience with the Paris system for reporting urinary cytology: Correlation of cytology and histology in 194 cases. Am. J. Clin. Pathol..

[39] Meilleroux J., Daniel G., Aziza J., d’Aure D.M., Quintyn-Ranty M.L., Basset C.M., Evrard S.M., Courtade-Saidi M.M. (2018). One year of experience using the Paris system for reporting urinary cytology. Cancer Cytopathol..

[40] Rohra P., Ocampo Gonzalez F.A., Yan L., Mir F., Furlan K., Basu S., Barua A., Cheng L., Park J.W. (2021). Effect of the Paris system for reporting urinary cytology with histologic follow-up. Diagn. Cytopathol..

[41] Ma C., Zhang L. (2020). Comparison of urine cytology diagnostic reports before and after the implementation of the Paris System classification system in China. Cytopathology.

[42] Roy M., Kaushal S., Jain D., Seth A., Iyer V.K., Mathur S.R. (2017). An institutional experience with The Paris System: A paradigm shift from ambiguous terminology to more objective criteria for reporting urine cytology. Cytopathology.

[43] Moulavasilis N., Lazaris A., Katafigiotis I., Stravodimos K., Constantinides C., Mikou P. (2020). Risk of malignancy assessment for the Paris system for reporting urinary cytology. Diagn. Cytopathol..

[44] VandenBussche C.J., Hang J.-F., McIntire P.J., Miki Y., Peyton S., Vohra P., Zhang M.L. (2022). Cytopathology of the upper urinary tract. The Paris System for Reporting Urinary Cytology.

[45] Zhang M.L., Miki Y., Hang J.F., Vohra M., Peyton S., McIntire P.J., VandenBussche C.J., Vohra P. (2021). A review of upper urinary tract cytology performance before and after the implementation of The Paris System. Cancer Cytopathol..

[46] Zhang M.L., VandenBussche C.J., Hang J.F., Miki Y., McIntire P.J., Peyton S., Vohra P. (2021). A review of urinary cytology in the setting of upper tract urothelial carcinoma. J. Am. Soc. Cytopathol..

[47] McIntire P.J., Snow J.T., Robinson B.D., Rao R.A., Goyal A., Heymann J.J., Siddiqui M.T. (2018). Improved correlation of urinary cytology specimens using The Paris System in biopsy-proven upper tract urothelial carcinomas. Cancer Cytopathol..

[48] Simon C.T., Skala S.L., Magers M.J., Weizer A., Kaffenberger S.D., Chinnaiyan A.M., Spratt D.E., Montgomery J., Mehra R., Lew M. (2019). The utility of upper urinary tract urine cytology before and after application of the Paris system. Diagn. Cytopathol..

[49] Xing J., Monaco S.E., Pantanowitz L. (2018). Utility of the Paris system for reporting urinary cytology in upper urinary tract specimens. J. Am. Soc. Cytopathol..

[50] Barkan G.A., Compton M.L., Elsheikh T.M., Ely K.A., Kurtycz D.F., Jorda M., Maleki Z., Minamiguchi S., Ohtani H., Piaton E. (2022). Atypical urothelial cells (AUC). The Paris System for Reporting Urinary Cytology.

[51] Brimo F., Auger M., Chebib I., Elsheikh T.M., Kinjo M., Piaton E., Pusztaszeri M., Shimokama T. (2022). Suspicious for high-grade urothelial carcinoma (SHGUC). The Paris System for Reporting Urinary Cytology.

[52] Siddiqui M.T., Allison D.B., Fadda G., Han J.-Y., McIntire P.J., Owens C.L., Tabatabai Z.L., Tsuzuki T., Zhang M.L. (2022). High-Grade Urothelial Carcinoma (HGUC). The Paris System for Reporting Urinary Cytology.

[53] Fite J.J., Rosenthal D.L., VandenBussche C.J. (2018). When words matter: A “suspicious” urinary tract cytology diagnosis improves patient follow-up among nonurologists. Cancer Cytopathol..

[54] Zhang M.L., Guo A.X., VandenBussche C.J. (2016). Morphologists overestimate the nuclear-to-cytoplasmic ratio. Cancer Cytopathol..

[55] Hang J.F., Charu V., Zhang M.L., VandenBussche C.J. (2017). Digital image analysis supports a nuclear-to-cytoplasmic ratio cutoff value of 0.5 for atypical urothelial cells. Cancer Cytopathol..

[56] Cowan M.L., VandenBussche C.J. (2018). The Paris system for reporting urinary cytology: Early review of the literature reveals successes and rare shortcomings. J. Am. Soc. Cytopathol..

[57] Hoda R.S., VandenBussche C., Hoda S.A. (2017). Diagnostic Liquid-Based Cytology.

[58] Hoda R.S., VandenBussche C., Hoda S.A. (2017). Urinary tract cytology. Diagnostic Liquid-Based Cytology.

[59] VandenBussche C.J., Rodriguez E.F., Allison D.B., Zhang M.L. (2019). Atlas of Cytopathology: A Pattern Based Approach.

[60] Nambirajan A., Jain D. (2018). Cell blocks in cytopathology: An update. Cytopathology.

[61] Wilson B.L., Russell D., Evans S.K., Agrawal T. (2021). Cell blocks in urine cytopathology: Do they add value to the diagnosis? A pilot study. J. Am. Soc. Cytopathol..

[62] Dantey K., Pantanowitz L., Xing J., Cuda J., Nestler R., Monaco S.E. (2019). Cell block preparation in urine cytology: Examination of utility and workflow in an academic practice. J. Am. Soc. Cytopathol..

[63] Barkan G.A., Tabatabai Z.L., Kurtycz D.F.I., Padmanabhan V., Souers R.J., Nayar R., Sturgis C.D. (2020). Practice patterns in urinary cytopathology prior to the Paris system for reporting urinary cytology. Arch. Pathol. Lab. Med..

[64] Chan E., Balassanian R., Tabatabai Z.L., Lou H., Vohra P. (2018). Improved diagnostic precision of urine cytology by implementation of The Paris System and the use of cell blocks. Cancer Cytopathol..

[65] Brisuda A., Hacek J., Cechova M., Skapa P., Babjuk M. (2018). Clinical and cytopathological factors affecting the cellularity of urinary cell blocks and the implication for diagnosis and follow-up of urinary bladder urothelial carcinoma. Cytopathology.

[66] Mansy S.S., Abbas M.A., Yehia H.A., Abdelrazik S.M., Ghanem L.Y., Amin T.M. (2006). Value of the innovated technique agarose cell block in improving the sensitivity of urine cytology in cases of bladder carcinoma. Ultrastruct. Pathol..

[67] Allison D.B., VandenBussche C.J. (2020). A review of urine ancillary tests in the era of the paris system. Acta Cytol..

[68] Qamar I., Rehman S., Mehdi G., Maheshwari V., Ansari H.A., Chauhan S. (2018). Utility of cytospin and cell block technology in evaluation of body fluids and urine samples: A comparative study. J. Cytol..

[69] Arville B., O’Rourke E., Chung F., Amin M., Bose S. (2013). Evaluation of a triple combination of cytokeratin 20, p53 and CD44 for improving detection of urothelial carcinoma in urine cytology specimens. Cytojournal.

[70] Renshaw A.A. (2006). Comparison of ureteral washing and biopsy specimens in the community setting. Cancer.

[71] Sheridan T.B., Walavalkar V., Yates J.K., Owens C.L., Fischer A.H. (2020). Cytologic processing of ureteral microbiopsies is associated with higher sensitivity for detection of urothelial carcinoma compared with conventional biopsy processing. J. Am. Soc. Cytopathol..

[72] Khan S., Omar T., Michelow P. (2012). Effectiveness of the cell block technique in diagnostic cytopathology. J. Cytol..

[73] Balassanian R., Wool G.D., Ono J.C., Olejnik-Nave J., Mah M.M., Sweeney B.J., Liberman H., Ljung B.M., Pitman M.B. (2016). A superior method for cell block preparation for fine-needle aspiration biopsies. Cancer Cytopathol..

[74] Springer S.U., Chen C.-H., Pena M.D.C.R., Li L., Douville C., Wang Y., Cohen J.D., Taheri D., Silliman N., Schaefer J. (2018). Non-invasive detection of urothelial cancer through the analysis of driver gene mutations and aneuploidy. eLife.

[75] Rodriguez Pena M.D.C., Springer S.U., Taheri D., Li L., Tregnago A.C., Eich M.L., Eltoum I.E.A., VandenBussche C.J., Papadopoulos N., Kinzler K.W. (2020). Performance of novel non-invasive urine assay UroSEEK in cohorts of equivocal urine cytology. Virchows Archiv.

